# Supplementary material for: Multi-objective optimal decision for orderly power utilization based on improved ε-constraint method in active distribution networks
Source: PLoS One. 2024 Oct 24;19(10):e0309437. doi: 10.1371/journal.pone.0309437 (PMC11500852; doi:10.1371/journal.pone.0309437)
Supplement: Attachment — Submitted filename: Response to Reviewers.docx [file pone.0309437.s002.docx]

March 15, 2024

Editorial office of Public Library of Science ONE

**Subject: Resubmission of the revised paper for review**

Dear Editorial office of Public Library of Science ONE,

We are thankful to the editors for their consideration and effort in evaluating our manuscript entitled “Multi-objective Optimal Decision for Orderly Power Utilization Based on Improved *ε*-Constraint Method” (PONE-D-23-41665). Please find below detailed responses to all reviewers' questions and concerns, as well as an explanation of how these were taken into consideration when revising the manuscript.

Yours sincerely,

Wen Xin, Li Hui and co-authors

**Authors’ Responses to the Comments on PONE-D-23-41665**

Wen Xin^1^, Li Hui^1^, Wu Xiaoqiang^1^, Li Yiwei^1^, Liu Siliang^2^, Huang Guohua^2^

March 15, 2024

The authors would like to thank the editor and reviewers for their comments and valuable suggestions that have helped to improve the quality of this manuscript. A response was prepared to address each of the concerns raised by the editor and reviewers. In order to identify each concern and correlate it with our response, we reproduced the editor’s and reviewers’ comments in underlined and italic font. We provided our response in normal font.

RESPONSE TO THE REVIEWERS

Reviewer(s)' Comments to Author:

**Reviewer: 1**

Comments to the Author

1. *English should be improved.*

**Response:** Thanks for your valuable and thoughtful comments. We have carefully checked and improved the English writing in the revised manuscript.

2. *Title: Please use the literature background to develop a better title in order to catch the prospective reader's attention.*

**Response:** Thanks for your valuable suggestion. In order to catch the prospective reader's attention, we have revised the title to Multi-objective Optimal Decision for Orderly Power Utilization Based on Improved *ε*-Constraint Method in Active Distribution Networks.

Please see the lines 1-3 of page 1 and remarks in **red**.

3. *Abstract: It should be rewritten in terms of aim, background, motivation, and significant results. Your abstract should clearly state the essence of the problem you are addressing, what you did, and what you found and recommended. That would help prospective readers of the abstract decide if they wish to read the entire article.*

**Response:** Thanks for your valuable suggestion. According to your suggestion, we have revised the abstract section in the manuscript, as follows:

“With the increasing demand for electricity load in China, orderly power utilization are important measures to alleviate electricity shortages during peak periods. This article establishes a multi-objective optimization model for orderly power utilization in active distribution networks is established, with the optimization objectives of minimizing the total operation cost, minimizing the cost for users, and minimizing the load fluctuation of the system. This model contains a large number of integer variables and nonlinear constraints, which is difficult to solve. To reduce computation time, convex relaxation techniques are adopted to transform the original model into a mixed-integer second-order cone programming (MISOCP) model, which has lower computational complexity. Furthermore, The improved *ε*-constraint method is proposed to solve the model, which can directly and quickly find the compromise optimal solution of the multi-objective problem. By using simplex search method, the proposed method dose not need to traverse all grid points, which can significantly reduce computation time. Finally, case study on the the IEEE-33 bus distribution network demonstrate the effectiveness of the proposed method.”

Please see the lines 12-22 of page 2 and remarks in **red**.

4. *The main concern I have about the paper is with respect to the contributions of your work. The methodology used has few outstanding innovation points. So what innovative work have you done compared with previous studies?*

**Response:** Thank you for your careful review of our paper. Our work made innovation in the model and the solution method. To clarify the innovation points more clearly, we have revised the last paragraph in the Introduction section, as follows:

“In summary, this paper makes two contribution: 1) A multi-objective optimization model for orderly power utilization in active distribution networks is established, with the optimization objectives of minimizing the total operation cost, minimizing the cost for users, and minimizing the load fluctuation of the system. For the nonlinear constraints in the model, convex relaxation techniques are adopted to transform the original model into a mixed-integer second-order cone programming (MISOCP) model, which has lower computational complexity. 2) The improved *ε*-constraint method is proposed to solve the model, which can directly and quickly find the compromise optimal solution of the multi-objective problem. Compared to the traditional *ε*-constraint method, the proposed method dose not need to traverse all grid points by using simplex search method, which can significantly reduce computation time.”

Please see the lines 54-61 of page 3 and remarks in **red**.

5. *How does your work contribute to this field? It is not clearly stated in the abstract and conclusion sections.*

**Response:** Thank you for your careful review of our paper. To clarify the contribution of our work in this to We have revised the abstract and conclusion sections in the manuscript, as follows:

“With the increasing demand for electricity load in China, orderly power utilization are important measures to alleviate electricity shortages during peak periods. This article establishes a multi-objective optimization model for orderly power utilization in active distribution networks is established, with the optimization objectives of minimizing the total operation cost, minimizing the cost for users, and minimizing the load fluctuation of the system. This model contains a large number of integer variables and nonlinear constraints, which is difficult to solve. To reduce computation time, convex relaxation techniques are adopted to transform the original model into a mixed-integer second-order cone programming (MISOCP) model, which has lower computational complexity. Furthermore, The improved *ε*-constraint method is proposed to solve the model, which can directly and quickly find the compromise optimal solution of the multi-objective problem. By using simplex search method, the proposed method dose not need to traverse all grid points, which can significantly reduce computation time. Finally, case study on the the IEEE-33 bus distribution network demonstrate the effectiveness of the proposed method.”

“This paper establishes a multi-objective optimization model for orderly power utilization in active distribution networks. Convex relaxation techniques are adopted to transform the original model into a MISOCP model, and the improved *ε*-constraint method is proposed to solve the model efficiently. Compared with the traditional single objective optimization model, the solution of multi-objective optimization is a solution with a high degree of comprehensive optimization. Compared with the traditional *ε*-constraint method, the objective value of the proposed method has been improved by 15.9% and the computation time is significantly reduced by 92.4% in the case studies.”

Please see the 12-22 of page 2, lines 352-357 of page 19 and remarks in **red**.

6. *Introduction: it is not well organized. With the current introduction, it is not possible to understand the gap between the previous research and the novelty of the current works. The introduction should be fully reorganized to show the difference between the current work and the previous one.*

**Response:** Thank you for your careful review of our paper. To show the difference between the current work and the previous one more clearly, we have revised the literature review and the last paragraph in the Introduction section in the manuscript, as follows:

“At present, there have been numerous scholars to carry out research on the decision-making of orderly power utilization. Peng et al. [4] establish the objective function by summing up the compensation cost for orderly power utilization methods and the cost associated with network loss penalties. By incorporating the network loss penalty term, they prioritize the utilization of power sources during the decision-making process for orderly power utilization. Zhang et al. [5] formulate a decision-making model for orderly power utilization, which coordinates both weekly and daily time scales. The objective of this model is to minimize the combined cost of peak avoidance and the penalty associated with fluctuations in peak and valley loads.The above literature are all single objective optimization model, both of them only consider the control operation cost of orderly power utilization, but not the impact of orderly power utilization measures on users. Contrasting the single objective optimization model, the multi-objective optimization model offers numerous non-dominated solutions, also known as the Pareto optimal solutions. The decision-maker need carefully choose a compromise optimal solution from among these Pareto optimal solutions. The approaches to solving the multi-objective optimization model can be categorized into heuristic optimization methods [6-7] and traditional optimization techniques [8-9]. Heuristic optimization methods offer the flexibility of directly solving the model without the limitations on its characteristics. Nevertheless, their computational inefficiency renders them impractical for real-world distribution networks. As for the traditional optimization methods, they mainly include the weighted sum method and the *ε*-constraint method. When using traditional optimization methods, there are usually restrictions on the type of model, and it is necessary to perform convex relaxation on the model. Wang et al. [8] propose a multi-objective optimization decision model for orderly power utilization and adopt the weighted sum method to solve the proposed model. The model aims at taking maximizing the grid load rate and maximizing the energy efficiency of power utilization. Paterakis et al. [9] adopt the *ε*-constraint method to solve a multi-objective optimization model, which aims to determine the optimal energy and reserve volumes considering the joint energy and reserve day-ahead market. The *ε*-constraint method divides the range of values of *ε_i_* into *q*-equal segments and generates distributed grid points. By traversing all grid points, the set of Pareto optimal solutions can be obtained. As the value of *q* increases, there will be more grid points, which will seriously affects the computation efficiency.

In summary, this paper makes two contribution: 1) A multi-objective optimization model for orderly power utilization in active distribution networks with the optimization objectives of minimizing the total operation cost, minimizing the cost for users, and minimizing the load fluctuation of the system. For the nonlinear constraints in the model, convex relaxation techniques are adopted to transform the original model into a mixed-integer second-order cone programming (MISOCP) model, which has lower computational complexity. 2) The improved *ε*-constraint method is proposed to solve the model, which can directly and quickly find the compromise optimal solution of the multi-objective problem. Compared to the traditional *ε*-constraint method, the proposed method dose not need to traverse all grid points by using simplex search method, which can significantly reduce computation time.”

Please see the lines 31-61 of page 2-3 and remarks in **red**.

7. *What is the new devolved methodology? It is not clearly presented.*

**Response:** Thank you for your careful review of our paper. Please let me clarify the new devolved methodology more clearly. By using the traditional *ε*-constraint method, the original model Eq. (R1) can be transformed into a single objective optimization model Eq. (R2), as follows:

$\begin{matrix} \min\left\{f_{1}(\mathbf{x}),f_{2}(\mathbf{x}),f_{3}(\mathbf{x})\right\} \\ \text{s.t.}\left\{\begin{matrix} \mathbf{g}(\mathbf{x})=0 \\ \mathbf{h}(\mathbf{x})\leq0 \end{matrix}\right. \end{matrix}$ (R1)

$\begin{matrix} \min f_{1}(x) \\ \text{s.t.}\left\{\begin{matrix} g(x)=0 \\ h(x)\leq0 \\ \text{ }f_{2}(x)\leq\varepsilon_{2},\text{ }f_{3}(x)\leq\varepsilon_{3} \end{matrix}\right. \end{matrix}$ (R2)

Where, *ε_i_* is the upper bound of each objective function *f_i_*(***x***) except the main objective function. The traditional *ε*-constraint method divides the range of values of *ε_i_* into *q*-equal segments, resulting in uniformly distributed grid points, as shown in Fig. 1. Each grid point corresponds to a set of values of *ε_i_*, and solving Eq. (R2) with different values of *ε_i_* can obtain the corresponding Pareto optimal solution. By traversing all the uniformly distributed grid points, the set of Pareto optimal solutions can be obtained, from which a solution is selected as the compromise optimal solution of the multi-objective optimization problem.

Fig. 1 Grid point of the traditional *ε*-constraint method

Obviously, as the value of *q* increases, there will be more grid points, which will seriously affect the computation efficiency. Therefore, we proposed a two-layer optimization model, as shown in Eq. (R3).

$\begin{matrix} \min_{\mu_{2},\mu_{3}} d\text{=}\sqrt{\overline{f}_{1}^{2}(x_{1}^{*})+\overline{f}_{2}^{2}(x_{1}^{*})+\overline{f}_{3}^{2}(x_{1}^{*})} \\ \text{s.t.}\left\{\begin{matrix} 0\leq\mu_{2}\leq1,\text{ 0}\leq\mu_{3}\leq1 \\ \mathbf{x}_{1}^{*}=\left\{\begin{matrix} \arg\min_{\mathbf{x}} \text{ }f_{1}\text{(}\mathbf{x}\text{)} \\ \text{s.t.}\left\{\begin{matrix} \mathbf{g}(\mathbf{x})\text{=0} \\ \mathbf{h}(\mathbf{x})\leq0 \\ \varepsilon_{2}=f_{2\min}+\mu_{2}(f_{2\max}-f_{2\min}) \\ \varepsilon_{3}=f_{3\min}+\mu_{3}(f_{3\max}-f_{3\min}) \\ f_{2}(\mathbf{x})\leq\varepsilon_{2},\text{ }f_{3}(\mathbf{x})\leq\varepsilon_{3} \end{matrix}\right. \end{matrix}\right. \end{matrix}\right. \end{matrix}$ (R3)

In the inner layer optimization model, auxiliary variables *μ*_2_ and *μ*_3_ are introduced and satisfy *μ*_2_, *μ*_3_∈[0,1], then the values of *ε*_2_ and *ε*_3_ can be uniquely determined by *μ*_2_ and *μ*_3_. Each time the inner layer optimization model is solved, the Pareto optimal solution corresponding to a certain set of auxiliary variables (*μ*_2_, *μ*_3_) is obtained and this solution is transferred to the outer layer optimization model. In the outer layer optimization model, each objective function is normalized to avoid the influence caused by the value range, as shown in Eq. (R4). The optimal values of the three objectives (*f_1_*_min_,*f_2_*_min_,*f_3_*_min_) are denoted as Utopia points. Since the three objectives cannot achieve the optimal value at the same time, the Utopia point is an unattainable ideal optimal solution. If the Euclidean distance between a solution and the Utopia point is smaller than other solutions, it can be considered as the highest degree of comprehensive optimal solution. Therefore, the objective function of the outer optimization model is to find a set of (*μ*_2_, *μ*_3_) that minimizes the Euclidean distance between the corresponding Pareto solution and the Utopia point (*f_1_*_min_,*f_2_*_min_,*f_3_*_min_). In this paper, a simplex search algorithm [16] is used to directly search for the optimal set of (*μ*_2_, *μ*_3_) within the range of values of *μ*_2_ and *μ*_3_. By solving the two-layer optimization model, the comprehensive optimal solution can be obtained directly, and it is no longer necessary to traverse every grid points. Therefore, the computation efficiency of the proposed method is significantly improved.

$\overline{f_{i}}=\frac{f_{i}-f_{i\min}}{f_{i\max}-f_{i\min}}$ (R4)

8. *The provided results are limited, not convincing, and insufficient to publish in this quality journal.*

**Response:** Thanks for your valuable suggestion. We have added the details in the Case study section in the manuscript, as follows:

“The IEEE-33 bus system is used to demonstrate the effectiveness of the proposed method, the line parameters are shown in [17], as shown in Fig. 3. The used computer is a PC with an Intel(R) Core(TM) i7-12700 and 32GB of RAM, and the computing platforms are MATLAB 2018b and Gurobi 9.0.2. Bus 18 and bus 30 are connected to photovoltaic power plant and wind farm, and the maximum active output is 6MW and 8MW, respectively. The predicted value of active output is shown in Fig. 4. Bus 24 and bus 30 are connected to the energy storage station, and the capacity is 2.5 MW·h. The distribution network is connected to the main grid from bus 1, and by adjusting the power injected from the main grid to the distribution network, the results of the schedule of orderly power utilization can be analyzed under different power gaps. The active load pattern of each user is taken from eight typical load curves in [18]. The participation of each user in orderly power utilization is shown in Table 1. To ensure the order of the measures of orderly power utilization, the compensation cost coefficient of peak transferring, peak shifting, peak clipping are set as *λ_trans_* = 300 yuan/h, *λ_shift_* = 500 yuan/MW·h, *λ_clip_*= 700 yuan/MW·h. The penalty cost coefficient of renewable energy curtailment is set as *λ_RES_* = 200 yuan/MW·h. And the charging and discharging operation cost coefficient of the energy storage station is set as *λ_ES_* = 150 yuan/MW·h.”

Please see the lines 288-299 of page 15-16 and remarks in **red**.

9. *Discussion of the results should provide useful insights. The results should be further elaborated to display how they could be utilized in real-world situations. The authors should further develop critical assessment in the results discussion.*

**Response:** Thanks for your valuable suggestion. We have added some results discussion in the CASE STUDY section in the manuscript, as follows:

“Compared with the results in Table 2, it can be seen that the compromised optimal solution is closer to the single-objective optimal value of each objective, and it is a comprehensively optimized solution with a higher degree of optimization. By solving the proposed multi-objective optimization, an orderly power utilization schedule can be obtained. This schedule simultaneously reduces the total operation cost and the cost for users, which can improve the economic benefits of the system operation. Moreover, it reduces the fluctuation of system load in the distribution network and enhances the operation security of the system.”

“It can be seen that for the traditional *ε*-constraint method, the number of solutions in the Pareto solution set increases significantly as the number of equal parts increases. Although the obtained compromise optimal solution has higher degree of comprehensive optimization, the computation time also increases significantly. Compared with the traditional *ε*-constraint method, the CPU time of the improved *ε*-constraint method is smaller. Based on several initial points, the proposed method generates the simplex in the feasible solution space and obtain the compromise optimal solution, which avoids solving a large number of Pareto solutions corresponding to all the mesh points. Therefore, the computation efficiency can be significantly improved. In addition, compared with the traditional *ε*-constraint method, the value of each objective function is better and the distance *d* between the compromise optimal solution and the Utopia point is also smaller. This is because the direct search algorithm takes points continuously in the feasible solution space, while the traditional algorithm takes points discretely in a grid, which is easy to miss the more optimized solutions existing between the discrete points. In summary, the proposed method significantly reduces computation time while obtaining a better compromise optimal solution.”

Please see the lines 316-319 of page 17, lines 330-338 of page 17 and remarks in **red**.

10. *Conclusions: It is poorly written and lacks an evaluation of the significant and main findings from the current work that have been found.*

**Response:** Thanks for your valuable suggestion. According to your suggestion, we have revised the Conclusions section in the manuscript, as follows:

“This paper establishes a multi-objective optimization model for orderly power utilization in active distribution networks. Convex relaxation techniques are adopted to transform the original model into a MISOCP model, and the improved *ε*-constraint method is proposed to solve the model efficiently. Compared with the traditional single objective optimization model, the solution of multi-objective optimization is a solution with a high degree of comprehensive optimization. Compared with the traditional *ε*-constraint method, the objective value of the proposed method has been improved by 15.9% and the computation time is significantly reduced by 92.4% in the case studies.”

Please see the lines 352-357 of page 19 and remarks in **red**.

**Reviewer: 2**

Comments to the Author

Authors proposed a multi-objective optimization to reduce the costs of electricity supply, including total operation costs, costs for the users, and minimization of system load fluctuations. Three demand response programs, such as peak transferring, peak shifting and peak clipping were considered. The work is interesting and relevant, however, some details should be clarified before publication.

1. *I would suggest extending and diversifying the literature review. A part of the paper contributions is the improved ε-constraint method, as there are several methods proposed based on the original method, it could be relevant to include them in the literature review and comment the advantages/suitability to the case study of the proposed algorithm.*

**Response:** Thanks for your valuable suggestion. According to your suggestion, we have revised the literature review undated the number of the references in the manuscript, as follows:

“At present, there have been numerous scholars to carry out research on the decision-making of orderly power utilization. Peng et al. [4] take the sum of the compensation cost of the means of orderly power utilization and the cost of network loss penalty as the objective function, and realizes the priority of calling the means which is beneficial to reduce the network loss in the decision-making of orderly power utilization by introducing the network loss penalty term. Zhang et al. [5] establish an orderly power use decision-making model under the coordination of weekly time scale and daily time scale, with the objective of minimizing the sum of the total cost of avoiding peaks and the penalty term of peak and valley differences.The above literatures are all single objective optimization model, both of them only consider the control operation cost of orderly power utilization, but not the impact of orderly power utilization measures on users. Different from the single objective optimization model, the multi-objective optimization model has multiple non-dominated solutions, and all non-dominated solutions constitute the Pareto optimal solutions. The decision maker needs to select a compromise optimal solution from the Pareto optimal solutions. The solution methods of multi-objective optimization model can be divided into heuristic optimization method [6-7] and traditional optimization method [8-9]. Heuristic optimization method can solve the model directly without some limitations on the model characteristics. However, they are not suitable for practical distribution networks due to their slow computational efficiency. Traditional optimization methods mainly include the weighted sum method and the *ε*-constraint method. Wang et al. [8] propose a multi-objective optimization decision model for orderly power utilization and adopt the weighted sum method to solve the proposed model. The model aims at taking maximizing the grid load rate and maximizing the energy efficiency of power utilization. Paterakis et al. [9] adopt the *ε*-constraint method to solve a multi-objective optimization model, which aims to determine the optimal energy and reserve volumes considering the joint energy and reserve day-ahead market. The *ε*-constraint method divides the range of values of *ε_i_* into *q*-equal segments and generates distributed grid points. By traversing all grid points, the set of Pareto optimal solutions can be obtained. As the value of *q* increases, there will be more grid points, which will seriously affects the computation efficiency.”

Please see the lines 31-51 of page 2-3 and remarks in **red**.

2. *Definitions of peak transferring and peak shifting looked very similar to me. If I did not misunderstand something, in both cases the loads are shifted in time, but the final total consumption does not change, and it looks like peak transferring is a particular case of peak shifting. If this is the case, could these two values be introduced as one? If not, could you better clarify the difference between the two elements?*

**Response:** Thank you for your careful review of our paper. As you said, the loads are shifted in both peak transferring and peak shifting case. However, the specific approach of these two measures is different. Peak transferring refers to transferring the total power load of users during a period by advancing or lagging time. And the peak shifting refers to shifting part of the power load of users from the peak period to the valley period. There are two main differences between them. Firstly, the starting time of peak transferring is unset, and the advancing or lagging time should be less than the maximum allowed time of peak transferring. But the time of peak period and valley period is set. Secondly, peak transferring will transfer the total power load of users, while peak shifting will shift part of power load. Therefore, the participation users of peak transferring and peak shifting are not the same, and these two values can not be introduced as one. In order to clarify the difference between peak transferring and peak shifting, we have revised the first paragraph in the Problem formulation section, as follows:

“Peak transferring refers to transferring the total power load of users during a period by advancing or lagging time.The starting time of peak transferring is unset, and the advancing or lagging time should be less than the maximum allowed time of peak transferring. Peak shifting refers to shifting part of the power load of users from the peak period to the valley period, and the time of peak period and valley period is set.”

Please see the lines 64-68 of page 4 and remarks in **red**.

3. *The choices of case study parameters (especially costs) should be stated and justified, for example, it is important to specify the discount rates applied to each element (peak transferring, peak shifting and peak clipping).*

**Response:** Thanks for your valuable suggestion. We have added the details of some parameters in the Case study section in the manuscript, as follows:

“The IEEE-33 bus system is used to demonstrate the effectiveness of the proposed method, the line parameters are shown in [17], as shown in Fig. 3. The used computer is a PC with an Intel(R) Core(TM) i7-12700 and 32GB of RAM, and the computing platforms are MATLAB 2018b and Gurobi 9.0.2. Bus 18 and bus 30 are connected to photovoltaic power plant and wind farm, and the maximum active output is 6MW and 8MW, respectively. The predicted value of active output is shown in Fig. 4. Bus 24 and bus 30 are connected to the energy storage station, and the capacity is 2.5 MW·h. The distribution network is connected to the main grid from bus 1, and by adjusting the power injected from the main grid to the distribution network, the results of the schedule of orderly power utilization can be analyzed under different power gaps. The active load pattern of each user is taken from eight typical load curves in [18]. The participation of each user in orderly power utilization is shown in Table 1. To ensure the order of the measures of orderly power utilization, the compensation cost coefficient of peak transferring, peak shifting, peak clipping are set as *λ_trans_* = 300 yuan/h, *λ_shift_* = 500 yuan/MW·h, *λ_clip_*= 700 yuan/MW·h. The penalty cost coefficient of renewable energy curtailment is set as *λ_RES_* = 200 yuan/MW·h. And the charging and discharging operation cost coefficient of the energy storage station is set as *λ_ES_* = 150 yuan/MW·h.”

Please see the lines 288-299 of page 15-16 and remarks in **red**.

4. *Also, the term “charging and discharging operation cost of the energy storage plant” is not clear to me. Why operation costs of batteries depend on the charging and discharging power at the time period?*

**Response:** Thank you for your careful review of our paper. The batteries’ lifetime in energy storage station will be decreased during the charging and discharging operation. For the convenience of calculating the depreciation cost during operation, we use the charging and discharging power.

5. *The whole text should be proofread to solve some typos. For example, confused words (“peak shifting, peak shifting, and peak clipping,…”, “staggering compensation coefficient”), unclear sentences structure (“in the feasible domain are selected. substituting…”), missing verbs in sentences. Variables in the equations and in the text must correspond (for example, utrans i,h, QLi,t). Also, location of legend in the Figure is confusing, it looks like ES, PV and WF are located at bus 33.*

**Response:** Thank you for your careful review of our paper. According to your suggestion, we have checked the whole text and made some revision in the manuscript, as follows:

“Among them, the compensation cost for orderly power utilization can be further divided into the compensation cost for peak transferring, peak shifting, and peak clipping, as shown below:”

“Where, *λ_clip_* is the compensation coefficient of peak clipping per unit time period, in yuan/MW·h.”

“three points ***X****_j_*(*μ*_2_,*μ*_3_) in the feasible solution space are selected, *j*=1,2,3.”

“Due to the serious impact of power cut on national economy and people's well being, this paper does not consider this measure.”

“*utrans i,h* is a 0-1 variable. When it takes the value of 1, it means that user *i* peak transferring *h* hours.”

“*ushift i,β* is a 0-1 variable, and when it takes the value of 1, it means that the *i*-th user participates in the *β*-th peak shifting. Δ*PLshift i,β,t* is the load correction corresponding to the *β*-th peak shift of user *i* at time *t*.”

“*C_i_* is the number of stages in which user *i* participates in peak clipping, and *ΔPclip i,t* is the amount of single-stage load modification in which user *i* participates in peak clipping at time *t*.”

“Δ*PLtrans i,t*, Δ*PLshift i,t* and Δ*PLclip i,t* are the load corrections for peak transferring, peak shifting and peak clipping for user *i* at time *t*, respectively.”

“In particular, when *utrans i,0*=1, it means that the user has 0 hour of time peak transferring.”

“Where, *ushift i,0*is a 0-1 variable that, when taking the value of 1, indicates that user *i* participates in peak shifting and when taking the value of 0, indicates that user *i* does not participate in peak shifting.”

“*uclip i,0* is a 0-1 variable that, when taking the value of 1, indicates that user *i* participates in peak clipping and when taking the value of 0, indicates that user *i* does not participate in peak clipping.”

“The IEEE-33 bus system is shown in Fig. 3, and the line parameters are shown in [17].

Fig. 3 IEEE-33 bus system

”

**Reviewer: 3**

Comments to the Author

This paper presents an approach to the optimal orderly power utilization problem using an enhanced epsilon-constraint optimization method. The authors should take into consideration the following issues:

1. *Page 2: sentence“Since power cut will seriously national production and life …” must be rephrased!*

**Response:** Thank you for your careful review of our paper. According to your suggestion, we have rephrased the sentence in the manuscript, as follows:

“Due to the serious impact of power cut on national economy and people's well being, this paper does not consider this measure.”

Please see the lines 70-71 of page 3 and remarks in **red**.

2. *Page 2: pay attention to repetitions: “the graded peak shifting and graded peak shifting”.*

**Response:** Thank you for your careful review of our paper. According to your suggestion, we have revised the repetitions in the manuscript, as follows:

“In this paper, the graded peak transferring and graded peak shifting are considered. ”

Please see the lines 76-77 of page 11 and remarks in **red**.

3. *Page 2, lines before eq. (1): again, pay attention to repetitions – “for peak shifting, peak shifting, and peak clipping”.*

**Response:** Thank you for your careful review of our paper. According to your suggestion, we have revised the repetitions in the manuscript, as follows:

“Among them, the compensation cost for orderly power utilization can be further divided into the compensation cost for peak transferring, peak shifting, and peak clipping, as shown below:”

Please see the lines 85-87 of page 11 and remarks in **red**.

4. *Page 3 and forward: when introducing different “lambda” coefficients, you should define their meaning, including units of measurement, e.g. lambda_trans, lambda_shift, lambda_clip, lambda_RES or lambda_ES. Basically, they should represents some kind of prices, but you should clearly define them.*

**Response:** Thanks for your valuable suggestion. According to your suggestion, we have clearly define the meaning and units of measurement of the “lamba” coefficients in the manuscript, as follows:

“*λ_trans_* is the compensation cost coefficient of peak transferring per unit time period, in yuan/h.”

“*λ_shift_* is the compensation cost coefficient of peak shifting per unit time period, in yuan/MW·h.”

“*λ_clip_* is the compensation coefficient of peak clipping per unit time period, in yuan/MW·h.”

“*λ_RES_* is the penalty cost coefficient of renewable energy curtailment, in yuan/MW·h.”

“*λ_ES_* is the charging and discharging operation cost coefficient of the energy storage plant, in yuan/MW·h.”

Please see the lines 95-115 of page 5-6 and remarks in **red**.

5. *Page 3 and forward: when use in text, different variables should use sub- and super-scripts, e.g. “utrans i,h” but other similar notations exist.*

**Response:** Thank you for your careful review of our paper. According your suggestion, we have revised the scripts of the variables in the text, as follows:

“*utrans i,h* is a 0-1 variable. When it takes the value of 1, it means that user *i* peak transferring *h* hours.”

“*ushift i,β* is a 0-1 variable, and when it takes the value of 1, it means that the *i*-th user participates in the *β*-th peak shifting. Δ*PLshift i,β,t* is the load correction corresponding to the *β*-th peak shift of user *i* at time *t*.”

“*C_i_* is the number of stages in which user *i* participates in peak clipping, and *ΔPclip i,t* is the amount of single-stage load modification in which user *i* participates in peak clipping at time *t*.”

“Δ*PLtrans i,t*, Δ*PLshift i,t* and Δ*PLclip i,t* are the load corrections for peak transferring, peak shifting and peak clipping for user *i* at time *t*, respectively.”

“In particular, when *utrans i,0*=1, it means that the user has 0 hour of time peak transferring.”

“Where, *ushift i,0*is a 0-1 variable that, when taking the value of 1, indicates that user *i* participates in peak shifting and when taking the value of 0, indicates that user *i* does not participate in peak shifting.”

“*uclip i,0* is a 0-1 variable that, when taking the value of 1, indicates that user *i* participates in peak clipping and when taking the value of 0, indicates that user *i* does not participate in peak clipping.”

6. *Page 3: as eq. (5) suggests, C_RES,t can be positive or negative, depending on the sign of differences between forecasted and scheduled output of photovoltaic and wind farms. Please, comment on this subject!*

**Response:** Thanks for your valuable suggestion. In Eq. (5), *P_sf,i,t_* and *P_wf,i,t_* are the predicted active output of photovoltaic power stations and wind farms at time *t*, respectively. And *P_s,i,t_* and *P_w,i,t_* are the scheduled output of photovoltaic power stations and wind farms at time *t*, respectively. The predicted output is always larger than the scheduled output, and the difference between them is the amount of renewable energy curtailment. Therefore, *C_RES,t_* would not be negative. In order to to clarify the problem more clearly, we have revised the explanation in the section “II. PROBLEM FORMULATION”, as follows:

“Where, *λ_RES_* is the penalty cost coefficient of renewable energy curtailment, in yuan/MW·h. *N_s_* and *N_w_* are the total number of photovoltaic power stations and wind farms, respectively. *P_sf,i,t_* and *P_wf,i,t_* are the predicted active output of photovoltaic power stations and wind farms at time *t*, respectively. And *P_s,i,t_* and *P_w,i,t_* are the scheduled output of photovoltaic power stations and wind farms at time *t*, respectively. The predicted output is always larger than the scheduled output, and the difference between them is the amount of renewable energy curtailment. ”

Please see the lines 110-114 of page 6 and remarks in **red**.

7. *Page 9, Step 5 of the algorithm: you refer to “the contraction formula (30)”. Please, check if the reference to eq. (30) is correct!*

**Response:** Thank you for your careful review of our paper. We have revised the number of the referenced formula in **Step 5** of the algorithm in the manuscript, as follow:

“**Step 4**: Calculate the reflection point ***X****_R_*. Calculate the reflection point ***X****_R_* according to the reflection Eq. (31) and check whether it is within the feasible solution space, i.e., whether it satisfies *μ*_2_, *μ*_3_∈[0,1]. If it is not in the feasible solution space, recalculate it according to the contraction Eq. (32) until a reflection point ***X****_R_* in the feasible solution space is obtained.

$\mathbf{X}_{R}=\mathbf{X}_{C}+K_{R}(\mathbf{X}_{C}-\mathbf{X}_{H})$ (31)

$\mathbf{X}_{R}=\mathbf{X}_{C}+K_{S}(\mathbf{X}_{R}-\mathbf{X}_{C})$ (32)

**Step 5**: Generate a new simplex. Calculate *d*(***X****_R_*) corresponding to the reflection point ***X****_R_* and compare *d*(***X****_R_*) with *d*(***X****_H_*) corresponding to the worst point ***X****_H_*. If *d*(***X****_R_*) > *d*(***X****_H_*), return to **Step 4** and calculate the new reflection point according to the contraction Eq. (32). ”

Please see the lines 278-286 of page 15 and remarks in **red**.

8. *Page 11, title of subsection D: explain what do you understand by “power gap”.*

**Response:** Thank you for your careful review of our paper. Power gap refers to a situation where the available electricity supply is insufficient to meet the demand in a specific region or at a particular time. This imbalance can be caused by various factors such as increased consumption, insufficient generation capacity, transmission and distribution issues, or even natural disasters.To make the description more accurate, we have revised “power gap” as “power shortage” in the manuscript, as follows:

“*D. Analysis of orderly power consumption under different power shortages*

In order to analyze the schedule of orderly power utilization under different power shortages, the proposed model are carried out by considering the existence of 5% and 15% power shortages in the peak period, respectively.

Tab. 5 Results of orderly power utilization under different power shortages

| Power shortage | Peak transferring/MWh | Peak shifting/MWh | Peak clipping/MWh |
| --- | --- | --- | --- |
| 5% | 27.3 | 13.4 | 0 |
| 10% | 33.5 | 29.7 | 8.7 |
| 15% | 34.8 | 37.1 | 28.6 |

”

Please see the lines 340-343 of page 18 and remarks in **red**.
